# An Investigation of Sleep Macro‐ and Microarchitecture by *APOE* Genotype

**DOI:** 10.1002/ana.78312

**Published:** 2026-09-22

**Authors:** Gawon Cho, Anne Chen, Eunyoung Choi, Orfeu M. Buxton, Daniel Kay, Adam Mecca, Brienne Miner

**Affiliations:** ^1^ Yale School of Medicine New Haven Connecticut USA; ^2^ Arizona State University Phoenix Arizona USA; ^3^ The Pennsylvania State University University Park Pennsylvania USA; ^4^ University of Rochester Medical Center Rochester New York USA

## Abstract

**Objectives:**

Apolipoprotein E ε4 (*APOE ε4*), a robust genetic risk factor for Alzheimer's disease (AD) is associated with functional connectivity deficits and amyloid pathology in brain regions involved in sleep regulation. Thus, alterations in sleep architecture may be one pathway through which *ε4* contributes to Alzheimer's disease vulnerability. However, sleep architecture characteristics associated with *APOE ε4* remain poorly characterized.

**Methods:**

This cross‐sectional study examined associations between *APOE* genotype and sleep architecture among adults aged 50–85 years enrolled in the Sleep Heart Health Study (N = 3,081). *APOE* genotype was classified into *ε4* heterozygotes, *ε4* homozygotes, *ε2* carriers, and *ε3* homozygotes (reference group). Sleep architecture was defined using macro‐level traits (percentage of time spent in in each sleep stage and wake after sleep onset) and micro‐level traits (spindle characteristics [power, density, and frequency], odds ratio product [ORP], and arousal index). Associations between *APOE* genotype and each sleep metric was evaluated using linear regression.

**Results:**

Macroarchitecture was largely comparable across genotypes. However, substantial differences in micro‐level traits were identified, including lower spindle power among *ε*4 homozygotes (*β* = −2.14, *p* = 0.02) and a dose–response decrease in spindle frequency at peak power with each *ε*4 count (*β*_*ε*4 heterozygotes_ = −0.04, *p* = 0.04; *β*_*ε*4 homozygote_ = −0.12, *p* = 0.065). Both ORP and arousal index decreased in a dose–response pattern with each *ε*4 count (ORP, *β*_*ε*4‐heterozygosity_ = −0.03, *β*_*ε*4‐homozygosity_ = −0.07, all *p* < 0.01; arousal index, *β*_*ε*4‐heterozygosity_ = −1.02, *p* = 0.006, *β*_*ε*4‐homozygosity_ = −1.78, *p* = 0.07). Between‐genotype differences in ORP widened with age. Associations did not vary by sex, race, cognitive status, pTau181, or A*β*42:40 ratio.

**Interpretation:**

Abnormal spindle activity observed among *ε*4 carriers may contribute to their elevated Alzheimer's disease risk. *ε4* carriers also showed less sleep fragmentation, which may reflect diminished arousability or electroencephalogram slowing due to neurodegenerative changes. ANN NEUROL 2026;100:1012–1022

Apolipoprotein E ε4 (*APOE ε4*) is a robust genetic risk factor for Alzheimer's disease (AD), a progressive neurodegenerative disorder affecting >7 million older adults in the US.^1^ *APOE ε4* carriers show reduced functional connectivity and greater amyloid burden in key subcortical neuromodulatory regions involved in sleep regulation, particularly the basal forebrain^2^, ^3^ and the locus coeruleus.^4^ These regions are central to sleep stage transitions that define sleep architecture, i.e., the organization of sleep period into stages of varying depth, including rapid eye movement (REM) sleep, non‐REM stages N1, N2, and N3 or slow‐wave sleep (SWS), as well as wakefulness.^5^, ^6^, ^7^, ^8^, ^9^ The locus coeruleus is also the primary source of norepinephrine in the central nervous system and modulates sleep spindles,^10^ brief bursts of rhythmic sigma‐band (11–16 Hz) brain activity that typically occur during N2 and contribute to memory consolidation.^11^, ^12^ Thus, greater susceptibility of these sleep‐regulating regions to neurodegenerative changes among *APOE ε4* carriers may lead to deficits in sleep architecture, such as sleep fragmentation and abnormal spindle activity, both of which are established markers of AD and related neurodegeneration.^13^, ^14^, ^15^, ^16^

To date, no study has examined differences in sleep microarchitecture by *APOE* genotype, including sleep spindles, despite their critical role in cognitive maintenance and AD pathogenesis.^11^, ^12^, ^16^ Moreover, to our knowledge, only two studies have evaluated differences in sleep macroarchitecture by *APOE* genotype, and they have reported heterogeneous findings.^17^, ^18^ Specifically, compared to non‐carriers, Tranah et al^17^ found male *APOE ε4* homozygotes to have more SWS and less sleep fragmentation, whereas André et al^18^ found adults carrying at least 1 *ε4* to have comparable SWS, but less REM sleep. Several features of these studies may have contributed to the heterogeneity in findings, and left important gaps unaddressed. Importantly, both studies relied on relatively smaller samples with limited representation of *ε4* homozygotes, more susceptible to outliers and limiting generalizability. In addition, the 2 studies focused on different and relatively narrow age ranges in late adulthood, and did not examine age‐related variations in the association between *APOE* genotype and sleep. This focus on late adulthood may also make genotype‐related differences difficult to detect, as sleep disturbances are highly prevalent in this life stage regardless of genetic risk.^19^, ^20^ Finally, only 1 of the 2 studies included women, despite their greater susceptibility to both AD and several forms of sleep disruption.^21^, ^22^, ^23^ *APOE ε4* also appears to confer greater AD risk in women,^22^, ^23^ underscoring the importance of characterizing sleep architecture in a sample inclusive of women.

To address these gaps, the present study examined associations between *APOE* genotype and sleep architecture, including both macro‐level and micro‐level traits, in a large, community‐based sample of middle‐aged and older adults, with robust representation of *APOE ε4* heterozygotes and homozygotes. Based on prior evidence showing reduced functional connectivity and greater amyloid pathology in sleep‐regulating brain regions among *APOE ε4* carriers,^2^, ^3^, ^4^ we hypothesized that they would show altered sleep architecture characterized by more sleep fragmentation (eg, less SWS and longer wake after sleep onset), less spindle activity, and less REM sleep relative to noncarriers. We further hypothesized that genotype‐associated differences would be more pronounced: (1) in middle age than in late adulthood, given the high prevalence of sleep disturbances later in life regardless of genetic risk; and (2) among women, given evidence showing *APOE ε4* to confer greater AD risk in women.^22^, ^23^ In addition to these hypothesis‐driven analyses, we also explored whether associations between *APOE* genotype and sleep architecture varied by race, cognitive status, and AD‐related pathology. These investigations represent an essential first step in assessing whether alterations in sleep architecture may serve as a modifiable risk factor for AD among *APOE ε4* carriers.

## Methods

### Study Sample

The Sleep Heart Health Study (SHHS) is a multicenter, community‐based cohort study designed to investigate relationships between sleep disorders and health outcomes in US adults. Details of the study design and data collection protocols have been described previously.^24^ Briefly, between 1995 and 1998, the SHHS enrolled 6,441 adults aged ≥40 years from several established parent cohorts, including the Atherosclerosis Risk in the Community Study (ARIC),^25^ the Cardiovascular Health Study (CHS),^26^ the Framingham Heart Study (FHS),^27^ and a few others. The enrolled participants subsequently completed a single night of unattended, in‐home overnight polysomnography.^24^ Blood samples were collected by the parent cohorts, facilitating *APOE* genotyping. Written informed consent was obtained at each SHHS or parent study visit, and ethical approvals were provided by institutional review boards of participating institutions, as described previously.^24^, ^25^, ^26^, ^27^

For the current study, we included participants who had complete information on polysomnography, *APOE* genotype, and covariates. Because *APOE* genotype was publicly available in only a subset of the parent cohorts (ie, ARIC, CHS, and FHS), our analytic sample was restricted to participants recruited from these 3 parent cohorts. The final analytic sample included 3,081 participants between ages 50 and 85 years (inclusive), after excluding individuals with missing data. See Figure S1 for processes used to derive the analytic sample. Secondary analyses of existing data were approved by the Yale University Institutional Review Board.

### Outcomes

Full‐montage in‐home polysomnography was performed using the Compumedics PS system following standardized protocols, including electroencephalography, electrooculography, chin electromyography, thoracic and abdominal plethysmography, airflow measurement, pulse oximetry, electrocardiography, and body position monitoring.^24^ Sleep stages were manually scored in 30‐second epochs, and arousals and respiratory events were identified according to standard criteria.^28^ Detailed procedures for polysomnography, data processing, and sleep scoring have been described previously.^24^, ^28^

The outcomes of interest included characteristics of macro‐ and micro‐level sleep architecture derived from this overnight in‐home polysomnography data. Macroarchitecture variables included the proportion of REM (%REM), the proportions of non‐REM stages (%N1, %N2, and %N3) and wake after sleep onset (WASO). Percentage of total sleep time spent in each sleep stage was calculated by dividing the total time spent in each stage by total sleep time. WASO was defined as total time spent awake in minutes between sleep onset and offset. Microarchitecture variables included N2 spindle characteristics (ie, density, power, and frequency), odds ratio product (ORP), and arousal index. Spindles during N2 were detected using a previously described method.^12^ Using the detected spindle events, spindle density was defined as the number of valid spindles per minute of N2 sleep, spindle power was defined as the average of peak spectral power across all valid spindles, and spindle frequency was defined as the electroencephalogram (EEG) frequency at which peak power occurred, averaged across spindles.^29^ ORP is a continuous measure of wake propensity ranging from 0 (always asleep) to 2.5 (always awake).^30^, ^31^, ^32^ ORP was calculated using established methods, in which the EEG power spectra in the delta, theta, alpha‐sigma, and beta bands for each 3‐second epoch were used to derive a ratio reflecting the likelihood that the epoch occurred during wakefulness based on a large reference dataset.^30^, ^31^, ^32^ ORP values for each 3‐second epoch were then averaged over total sleep time. Stage‐specific ORPs were calculated as well by taking the average across REM, N1, N2, and N3. The average ORP values represent mean wake propensity during the respective sleep period and are independent of conventional sleep staging.^30^, ^31^, ^32^ Arousal index was computed as the number of arousal events per hour of total sleep time.

In addition to sleep architecture, we examined alternative sleep characteristics, including total sleep time (total time spent asleep between sleep onset and offset), apnea‐hypopnea index using the 4% criteria (AHI4%), and Epworth Sleepiness Scale score.^33^

### Exposure

*APOE* carrier status was categorized into the following 4 groups based on genotyping performed using DNA extracted from blood: *ε4* heterozygotes (*ε2*/*ε4* or *ε3*/ε4), *ε4* homozygotes (*ε4*/*ε4*), *ε2* carriers (*ε2*/*ε2* or *ε2*/*ε3*), and *ε3* homozygotes (*ε3*/*ε3*). *APOE ε4* heterozygotes and homozygotes were analyzed as distinct groups to assess potential dose‐dependent effects of *ε4*. *APOE ε3* homozygotes served as the reference group, as they are the population majority.^21^, ^22^ Detailed protocols for blood collection, DNA isolation, and *APOE* genotyping have been described previously.^34^, ^35^, ^36^

### Covariates

Covariates included in the primary analyses were age, sex, race (white and black), marital status, educational attainment, parent cohort, cognitive status, hypertension, diabetes, body mass index, total sleep duration, AHI4%, and the use of sleeping pills. Age was mean‐centered to improve the interpretability of interaction terms involving age. Cognitive status was classified as probable dementia versus no dementia. For participants recruited from the CHS and FHS cohorts, probable dementia was defined using education‐stratified Mini‐Mental State Examination (MMSE) cutoffs: <27 for college graduates and <24 for individuals without a college degree.^37^ CHS participants completed the modified MMSE, and their scores were converted to MMSE using a previously published method.^38^ FHS participants completed the MMSE, and scores were prorated to account for items not completed for reasons unrelated to cognitive function, such as visual impairment or language barriers, following prior research.^39^ In ARIC, probable dementia was adjudicated by an expert panel based on comprehensive review of available data, including neuropsychological testing and medical records.^40^ Additional analyses examined the sensitivity of results to replacing probable dementia status with plasma pTau181 and A*β*42/40 ratio. Among the 3 parent cohorts, pTau181 data were available only from the CHS, where frozen specimens collected between 1992 and 1993 were assayed using single‐molecule array (Quanterix).^41^ Data on the A*β*42/40 ratio were available in both CHS and FHS. In CHS, A*β*40 and A*β*42 concentrations were measured using electrochemiluminescence (MESO Scale Diagnostics) from the same specimens used for pTau181.^41^ In FHS, specimens were collected between 1998 and 2001, and amyloid measurements were obtained using a fluorimetric immunoassay (INNO‐BIA plasma amyloid‐beta; Innogenetics).^42^

### Statistical Analysis

Sample characteristics were described using means along with standard deviations for continuous variables, and counts and proportions for categorical variables. Differences in sample characteristics by *APOE* genotype were assessed using ANOVA for continuous variables and χ^2^ for categorical variables. The primary analysis tested the association between *APOE* genotype and each sleep variable (including macro‐ and micro‐level traits) using linear regression models, adjusting for age, sex, race, marital status, educational attainment, parent cohort, cognitive status, hypertension, diabetes, body mass index, total sleep duration, AHI4%, and use of sleeping pills. Sensitivity of the primary analysis to excluding all *APOE ε2* carriers was examined by classifying *APOE* genotype into noncarriers (*ε3ε3*), heterozygotes (*ε3ε4*), and homozygotes (*ε4ε4*). In addition, we examined the interaction of *APOE* genotype with age, sex, race, cognitive status, pTau181, and A*β*42:40, respectively, by interacting each term with *APOE* genotype in separate models. For sleep characteristics showing a significant genotype‐by‐age interaction, recycled predictions were calculated across combinations of genotype and age to visualize age‐related trends within each genotype group. For analyses involving interactions with race, cognitive status, p‐Tau181, and A*β*42:40 ratio, respectively, *APOE ε4* heterozygotes and homozygotes were combined into a single group, because the numbers of *ε4* homozygotes who identified as a black person, had probable dementia, or had information on the blood biomarkers were limited. In addition to sleep architecture, differences in alternative sleep characteristics (ie, total sleep time, AHI4%, and Epworth Sleepiness Scale score) by *APOE* genotype were assessed using linear regression, adjusting for covariates. Statistical significance was defined at the level of *p* < 0.05. All analyses were conducted using Python 3.10 (Python Software Foundation).

## Results

### Sample Characteristics

Table 1 shows sample characteristics. Mean age was 66.7 years (standard deviation 8.8), 52.8% were women, and 94.9% were white. 83.7% were married and 35.0% completed ≥16 years of education. A total of 770 persons carried 1 *APOE ε4* allele and 66 carried 2 *APOE ε4* alleles. All covariates were comparable across *APOE* genotype, with the exception of race, parent cohort, and body mass index. Average sleep characteristics are summarized in Table S0. Average %REM and %N3 were 19.9% and 18.6%, respectively. Individuals had a mean of 19.5 arousals per hour.

**TABLE 1:** Sample Characteristics

Characteristics	N (%) or mean (standard deviation)^a^					
	Full sample (N = 3081)	*APOE* genotype				
		*E*4 heterozygotes^b^ (N = 770)	*E*4 homozygotes (N = 66)	E2 carriers (N = 411)	E3 homozygotes (N = 1834)	*p* ^c^
Age	66.7 (8.7)	66.6 (8.71)	66.1 (7.94)	66.8 (9.07)	66.7 (8.76)	0.91
Sex						0.39
Males	1455 (47.2)	353 (45.8)	29 (43.9)	184 (44.8)	889 (48.5)	
Females	1626 (52.8)	417 (54.2)	37 (56.1)	227 (55.2)	945 (51.5)	
Race						<0.01
White	2923 (94.9)	730 (94.8)	62 (93.9)	375 (91.2)	1756 (95.8)	
Non‐White	158 (5.1)	40 (5.2)	4 (6.1)	36 (8.8)	78 (4.3)	
Marital status						0.36
Married	2579 (83.7)	659 (85.6)	54 (81.8)	337 (82.0)	1529 (83.4)	
Not married	502 (16.3)	111 (14.4)	12 (18.2)	74 (18.0)	305 (16.6)	
Educational attainment						0.66
15 years or less	2004 (65.0)	499 (64.8)	41 (62.1)	258 (62.8)	1206 (65.8)	
16 years or longer	1077 (35.0)	271 (35.2)	25 (37.9)	153 (37.2)	628 (34.2)	
Dementia	68 (2.2)	25 (3.3)	3 (4.6)	7 (1.7)	33 (1.8)	0.06
Diabetes	242 (7.9)	45 (5.8)	3 (4.6)	35 (8.5)	159 (8.7)	0.06
Hypertension	1329 (43.1)	332 (43.1)	29 (43.9)	178 (43.3)	790 (43.1)	1.00
Body mass index	28.3 (4.86)	27.9 (4.69)	28.8 (5.44)	28.5 (4.92)	28.5 (4.88)	0.02
Total sleep time	360.9 (62.39)	360.2 (65.59)	357.3 (63.41)	359.8 (61.16)	361.5 (61.28)	0.89
AHI4%	10.5 (13.02)	10.9 (13.87)	10.3 (12.56)	10.7 (13.22)	10.3 (12.62)	0.68
Uses sleep medication	703 (22.8)	191 (24.8)	19 (28.8)	97 (23.6)	396 (21.6)	0.19
Epworth sleepiness scale	7.7 (4.28)	7.7 (4.25)	7.0 (3.98)	7.8 (4.40)	7.7 (4.27)	0.53
Parent cohort						<0.01
CHS	935 (30.4)	232 (30.1)	15 (22.7)	134 (32.6)	554 (30.2)	
FHS	528 (17.1)	106 (13.8)	5 (7.6)	71 (17.3)	346 (18.9)	
ARIC	1618 (52.5)	432 (56.1)	46 (69.7)	206 (50.1)	934 (50.9)	

^a^ N (%) is reported for categorical variables and mean (standard deviation) are reported for continuous variables. Estimates in each genotype group correspond to individuals with a given genotype.

^b^ Includes APOE ε4ε2 and ε4ε3 carriers.

^c^ Comparison of sample characteristics across *APOE* genotype used chi‐square tests for categorical variables and ANOVA for continuous variables.

Abbreviations: AHI4% = Apnea‐hypopnea index based on the 4% desaturation criterion; APOE = apolipoprotein E; ARIC = Atherosclerosis risk in the community study; CHS = Cardiovascular health study; FHS = Framingham heart study.

### Macro‐Level Sleep Traits

Figure 1 graphically shows associations between macro‐level sleep architecture and *APOE* genotype, estimated using models adjusted for all covariates (1A: time spent in each stage; 1B: WASO; Table S1 includes detailed estimates). In models without interaction terms, %REM, %N1, %N2, %N3, and WASO did not differ across the genotypes. Nonetheless, significant age‐by‐genotype interaction was found for WASO, such that mean WASO declined by 1.57 min with each year of age among *ε4* homozygotes compared with *ε3* homozygotes (*p* = 0.025). Accordingly, recycled predictions showed a decreasing trend in WASO across the examined age range among *ε4* homozygotes, whereas it increased monotonically among *APOE ε3* homozygotes (Fig 2A). Associations of %REM, %N1, %N2, and %N3 with *APOE* genotype did not vary with age. Moreover, associations between *APOE* genotype and macro sleep architecture generally did not vary by sex (Table S2), race (Table S3), cognitive status (Table S4), pTau181 (Table S5), and a*β*42:40 (Table S6) with a few exceptions. Interaction between sex and *APOE ε4* homozygosity was significant, such that *ε4* homozygote vs. *ε3* homozygote difference in %REM was 3.33 percentage point lower in women than that in men (interaction p=0.046). Interaction of *APOE ε4* genotype with a*β*42:40 ratio was also significant, where *ε4* carriers spent less time in N3 with each unit increase of A*β*42:40 (Table S6).

**FIGURE 1:**
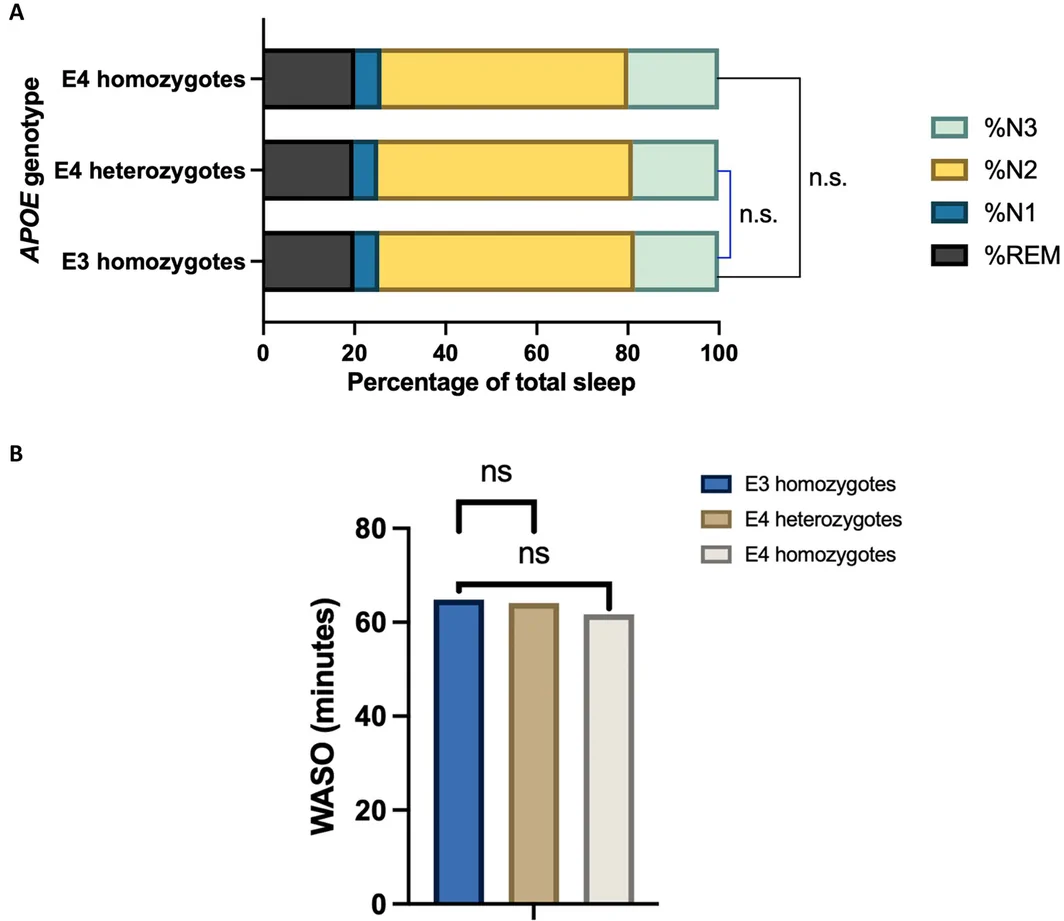
Association between macro‐level sleep architecture and *APOE* genotype. Bar height represents unadjusted genotype‐specific means for each sleep metric. Between‐genotype differences were derived from fully‐adjusted regression models. APOE = apolipoprotein E; REM = rapid eye movement. [Color figure can be viewed at www.annalsofneurology.org]

**FIGURE 2:**
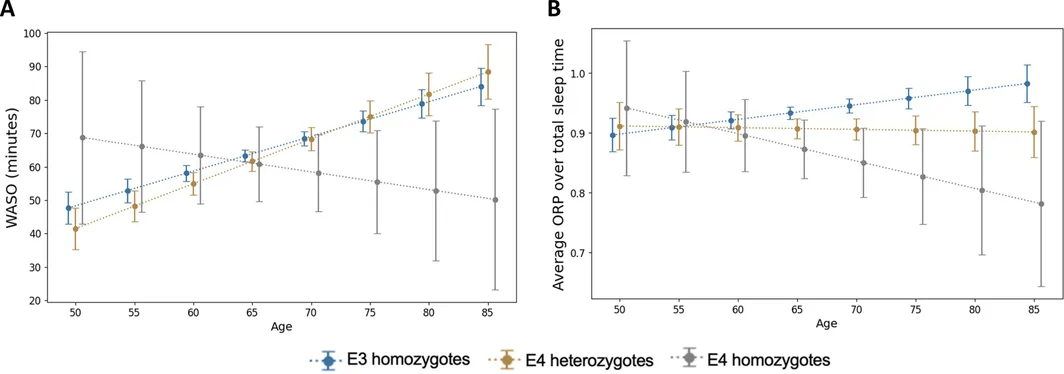
Differences in odds ratio product by apolipoprotein E genotype and age. ORP = odds ratio product; WASO = wake after sleep onset. [Color figure can be viewed at www.annalsofneurology.org]

### Micro‐Level Sleep Traits

Figure 3 shows associations between sleep spindle characteristics and *APOE* genotype, estimated using models adjusted for all covariates (Table S7 includes detailed estimates). In models without interaction terms, *ε4* homozygotes showed lower spindle power compared with *APOE ε3* homozygotes (Fig 3A; *β* = −2.14, *p* = 0.02). Frequency at the peak power showed a dose–response decrease with each *ε4* copy number that was significant among heterozygotes and borderline significant among homozygotes (Fig 3C; *β*_*ε4* heterozygotes_ = −0.044, *p* = 0.04; *β*_*ε4* homozygotes_ = −0.12, *p* = 0.065). *ε4* homozygotes also showed borderline significantly lower spindle density (Fig 3B; *β* = −0.14, *p* = 0.057). The association between sleep spindle characteristics and *APOE* genotype did not vary by age, as shown in Table S7. Investigation of ORP found a dose–response decrease in average ORP over total sleep time with each additional *APOE ε4* copy number (Fig 4A; *β*_*ε4* heterozygosity_ = −0.03, *β*_*ε4* homozygosity_ = −0.07, all *p* < 0.01; Table S8 includes detailed estimates). This dose–response pattern in ORP was replicated across all sleep stages (Fig 4B). Differences in ORP between *ε4* homozygotes and *ε3* homozygotes widened with advancing age during total sleep and N2, and differences between *ε4* heterozygotes and *ε3* homozygotes widened during total sleep, REM, and N1 (Table S8). Fig 2B graphically shows age‐related trends in average ORP over total sleep time by genotype, where *ε4* homozygotes show the steepest downward trend in ORP across the examined age range, *ε4* heterozygotes show a relative plateau, and *ε3* homozygotes show an upwards trend. In addition, arousal index showed a dose‐dependent decrease with *ε4* copy number that was significant among heterozygotes and borderline significant among homozygotes (Fig 5; *β*_heterozygotes_ = −1.02, *p* = 0.006; *β*_homozygotes_ = 1.78, *p* = 0.07). The association did not vary by age (Table S8). Associations between *APOE ε4* carrier status and micro sleep architecture did not vary by sex (Table S9), race (Table S10), cognitive status (Table S11), pTau181 (Table S12), and A*β*42:40 (Table S13).

**FIGURE 3:**
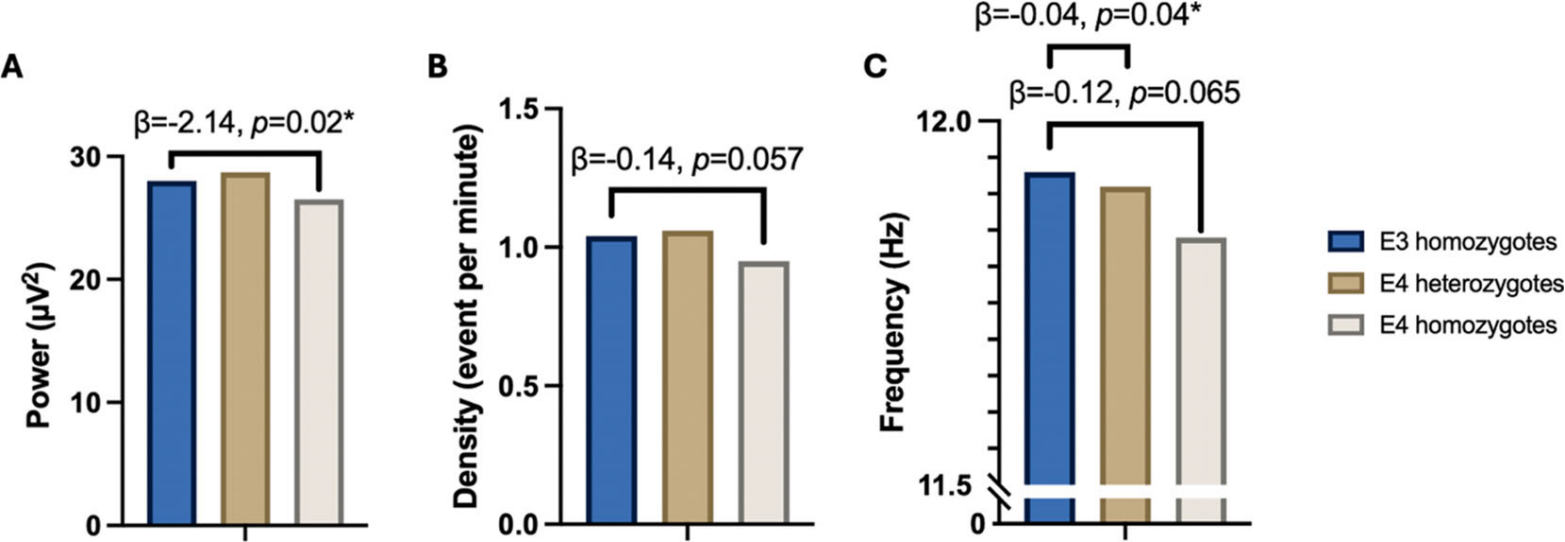
Association between sleep spindle activity and apolipoprotein E genotype. Bar height represents unadjusted genotype‐specific means for each sleep metric. Between‐genotype differences were derived from fully‐adjusted regression models. *Designates statistical significance of *β* from fully‐adjusted models at the *p* < 0.05 level. Non‐significant between‐genotype differences are not shown. [Color figure can be viewed at www.annalsofneurology.org]

**FIGURE 4:**
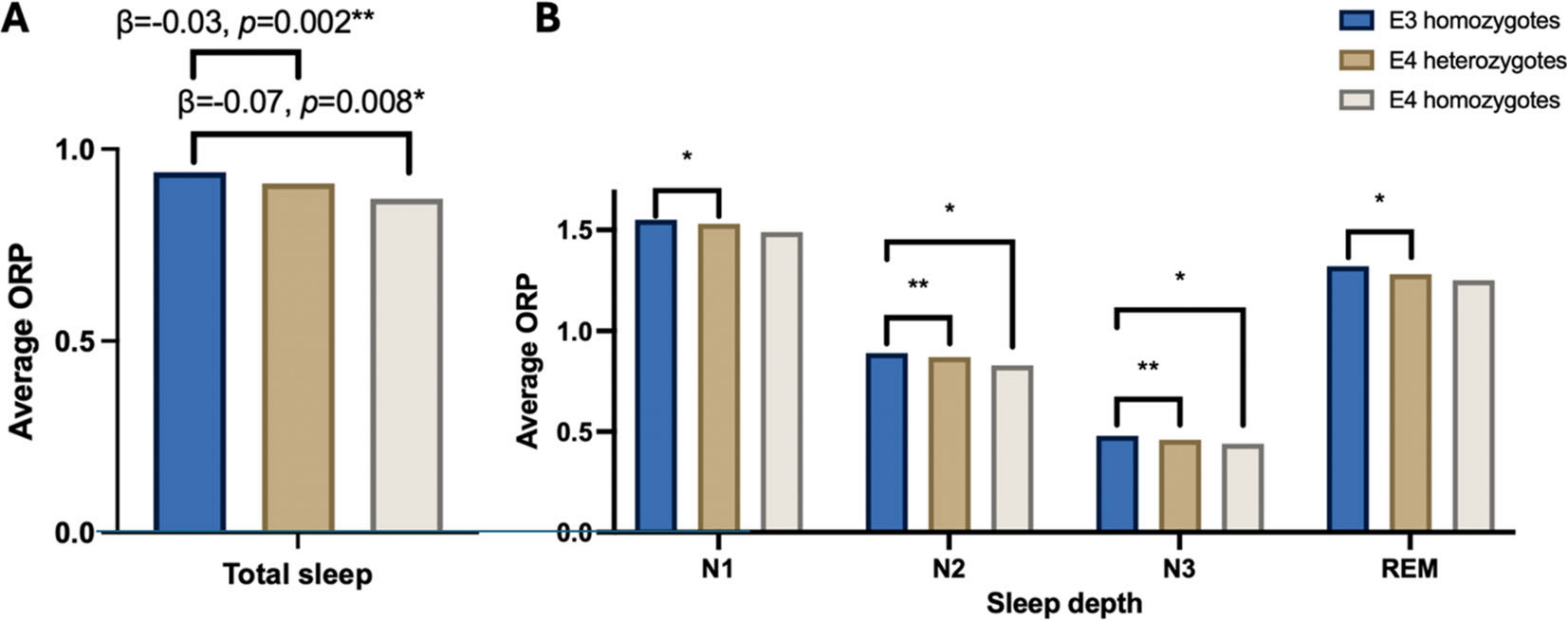
Association between odds ratio product and apolipoprotein E genotype. Bar height represents unadjusted genotype‐specific means for each sleep metric. Between‐genotype differences were derived from fully‐adjusted regression models. Stage‐specific average odds ratio product analyses were restricted to individuals with non‐zero total sleep time in the corresponding sleep stage. *Designates statistical significance of *β* from fully‐adjusted models at the *p* < 0.05 level. **Designates statistical significance of *β* from fully‐adjusted models at the *p* < 0.005 level. Non‐significant between‐genotype differences are not shown. ORP = odds ratio product; REM = rapid eye movement. [Color figure can be viewed at www.annalsofneurology.org]

**FIGURE 5:**
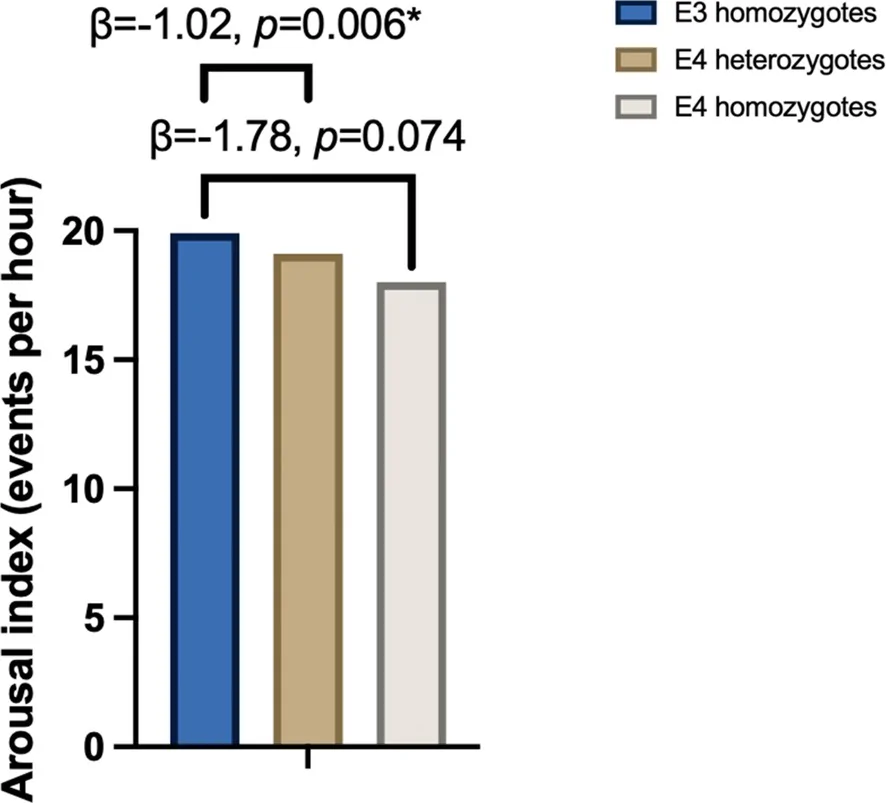
Association between arousal index and *APOE* genotype. Bar height represents unadjusted genotype‐specific means for each sleep metric. Between‐genotype differences were derived from fully‐adjusted regression models. *Designates statistical significance of *β* from fully‐adjusted models at the *p* < 0.05 level. **Designates statistical significance of *β* from fully‐adjusted models at the *p* < 0.005 level. Non‐significant between‐genotype differences are not shown. APOE = Apolipoprotein E. [Color figure can be viewed at www.annalsofneurology.org]

The alternative sleep characteristics, including total sleep time, AHI4%, and Epworth Sleepiness Scale score, generally did not differ by *APOE* genotype after adjustment for covariates (Table S14), although AHI4% was slightly elevated in *ε4* heterozygotes (*β* = 1.30, *p* = 0.02), but not in *ε4* homozygotes. Associations of *APOE* genotype with macro‐ and micro‐level traits were largely robust to the exclusion of all *ε2* carriers, although estimates for spindle characteristics were slightly attenuated (Table S15). Associations of *APOE* genotype with macro‐ and microarchitectural characteristics were also robust to adjustments for the burden of sleep apnea using AHI3% instead of AHI4% (Table S16).

## Discussion

This study aimed to investigate whether sleep architecture may serve as a pathway to elevated AD risk among *APOE ε4* carriers, through a comprehensive investigation of macro‐ and micro‐level characteristic. We had hypothesized that *APOE ε4* carriers would show alterations in sleep architecture that have been robustly associated with neurodegeneration, including abnormal spindle activity.^11^, ^12^ Supporting this hypothesis, significant deficits in sleep spindle characteristics were found even after adjustment for sociodemographic characteristics, sleep‐disordered breathing, sleep duration, sleeping pill use, and medical history, including diabetes, hypertension, and obesity. Specifically, *ε4* homozygotes showed less N2 spindle power compared with *ε3* homozygotes. Spindle frequency at peak power showed a dose‐dependent decrease, suggesting spindle slowing with increasing *ε4* copy number. *ε4* homozygotes also showed a borderline significant reduction in spindle density.

Sleep spindles play a key role in hippocampal–cortical communication necessary for memory consolidation,^43^ and reductions in spindle density and power have been robustly associated with AD and cognitive aging.^11^, ^12^, ^44^, ^45^ Our study extends this line of literature by showing that *APOE ε4* carriers, especially homozygotes who carry the highest genetic risk for AD, show abnormal spindle patterns consistent with AD pathogenesis or related neurodegenerative changes.^11^, ^12^, ^44^, ^45^ Abnormal spindle activity may in turn contribute to further AD pathogenesis by diminishing the neuroprotective effects of sleep spindles.^10^, ^43^ Thus, sleep spindles may constitute a pathway linking *APOE ε4* to AD, although causal conclusions cannot be drawn from this observational study. To our knowledge, this is the first study to examine differences in spindle activity by *APOE* genotype.

The current study also hypothesized that *APOE ε4* carriers would show greater sleep fragmentation. Contrary to this hypothesis, ORP—an EEG‐based measure of wake propensity where lower values reflect reduced wakefulness during nocturnal sleep^30^, ^31^, ^32^—decreased in a dose‐dependent manner with each *ε4* copy number during total sleep time and across all sleep stages. Age‐stratified analyses further revealed divergent trajectories by genotype. *ε3* homozygotes showed a monotonic increase in ORP with age, resembling previously documented sleep fragmentation trends in normal aging,^11^, ^12^ whereas *ε4* heterozygotes showed a plateau, and *ε4* homozygotes showed a downward trend with age. Patterns observed for ORP were generally consistent with arousal index and WASO, collectively suggesting that *APOE ε4* carriers have lower levels of sleep fragmentation than *ε3* homozygotes in middle and late adulthood.

Lower sleep fragmentation among *APOE ε4* carriers was unexpected, given the well‐established association of increased sleep fragmentation with AD and cognitive impairment.^46^, ^47^ Nonetheless, our findings are consistent with Tranah et al, who reported a dose‐dependent reduction in WASO with increasing *ε4* count.^17^ More recent studies have also reported mixed associations between sleep fragmentation measures, including WASO and arousals, and AD pathogenesis.^15^, ^48^ Emerging evidence on the glymphatic system, a perivascular brain waste clearance pathway, further suggests that some arousals may be temporally linked to enhanced cerebrospinal fluid movement involved in waste clearance and therefore be beneficial.^10^ Together, the mixed associations observed among sleep fragmentation, cognitive health, and *APOE* genotype raise the possibility that, in addition to excessive sleep fragmentation, markedly reduced sleep fragmentation may also be maladaptive, suggesting an optimal “sweet spot” for sleep continuity.

“EEG slowing” may provide one potential explanation for why lower wake propensity, fewer arousals, and shorter WASO among APOE *ε4* carriers may be maladaptive. EEG slowing is defined as a global low‐frequency shift in EEG activity, including intrusions of delta band activity into wakefulness and REM sleep, as well as reductions in alpha and sigma band activity during REM and NREM sleep.^49^, ^50^, ^51^ EEG slowing has been robustly associated with gray matter atrophy, AD, and mild cognitive impairment in human adults.^49^, ^50^, ^51^ Experimental work has further shown that animal models of cortical deafferentation induced by surgical transection show EEG patterns typically associated with deep sleep, such as increased delta‐band activity and prolonged hyperpolarization, during wakefulness and REM sleep.^52^ Lower levels of sleep fragmentation and wake propensity among *APOE ε4* carriers may reflect this low‐frequency shift of EEG activity consistent with EEG slowing, potentially due to greater underlying burden of neurodegeneration. In particular, the locus coeruleus and basal forebrain are wake‐promoting regions involved in sleep regulation and are vulnerable to early AD‐related pathogenesis.^2^, ^3^, ^4^ Neurodegeneration in these regions may therefore contribute to reductions in wake‐like, high‐frequency EEG activity. Future research should directly examine whether atrophy in subcortical neuromodulatory regions blunts cortical activation and higher‐frequency EEG activity required for arousals and awakenings.^6^, ^7^, ^8^, ^9^

Finally, *ε4* carriers showed an incremental decrease in SWS with each 1‐unit increase in A*β*42:40 ratio compared with *ε3* homozygotes. These findings suggest that *ε4* carriers may have less SWS than *APOE ε3* homozygotes when AD burden is absent or less advanced, whereas this difference appears to diminish with increasing AD burden. Interestingly, plasma pTau181 did not modify associations between *APOE* genotype and any of the macro‐ or microarchitectural characteristics. Given that amyloid accumulation may precede detectable elevations in Tau burden,^53^ deficits in SWS may be more apparent during the earliest preclinical phases of AD. Further research is needed to examine how the association between *APOE* genotype and sleep architecture change across the AD spectrum.

Strengths of the current study include the following. First, to our knowledge, this study is the first to examine differences in sleep microarchitecture by *APOE* genotype, identifying sleep signatures at the micro‐time scale that may be indicative of neurodegeneration among *ε4* carriers. Second, this study used gold standard polysomnography to comprehensively assess multiple features of sleep architecture that have been linked to AD risk.^14^, ^44^, ^45^ Third, this study included the largest number of *APOE ε4* heterozygotes and homozygotes among existing studies on this topic, and focused on community‐dwelling adults, thereby enhancing the applicability and generalizability of the findings. Fourth, the inclusion of middle‐aged adults allowed for the examination of age‐related trends in the association between *APOE* genotype and sleep. Fifth, this study comprehensively explored differences in associations between sleep architecture and *APOE* genotype by cognitive status, AD pathology, race, and sex. Sixth, all models were adjusted for AHI4% and total sleep time, making genotype‐related differences found in this study unlikely to be attributable to underlying obstructive sleep apnea or abnormal sleep duration. Also, *ε4* homozygotes showed comparable AHI4% with *ε3* homozygotes, in line with prior systematic reviews and meta‐analyses that have likewise found no clear association between *APOE* genotype and sleep disordered breathing.^54^ Seventh, our findings are likely to be robust against survival bias. Although prior research has shown higher overall mortality among *APOE ε4* carriers,^55^ our supplementary analyses of survival probability by *APOE* genotype, shown in Figure S2, indicated that survival probability of *ε4* heterozygotes and homozygotes was comparable to that of *ε3* homozygotes past the age span examined in this study.

Nonetheless, this study has several limitations. First, polysomnography assesses nocturnal sleep and does not capture certain features of sleep health that may be relevant for AD risk, such as daytime napping behavior.^56^ Second, we did not examine microstructural features beyond arousals, ORP, and sleep spindles. Third, sleep architecture was assessed based on a single night of polysomnography, which may be influenced by the “first night effect,” a well‐documented phenomenon wherein sleep is disturbed during the initial night of monitoring.^57^ As a result, our estimates of sleep architecture may not fully reflect individuals' habitual sleep patterns. Fourth, >90% of the analytic sample identified as white, which may limit the generalizability of our results to other racial and ethnic groups. Future studies should address these limitations by examining the association between *APOE* genotype and sleep using more comprehensive assessments, including measures of daytime sleep health, in diverse populations.

## Conclusions

This study conducted a comprehensive investigation of differences in macro‐ and micro‐level sleep architecture by *APOE* genotype in a large population‐based sample of middle‐aged and older adults. Compared with *APOE ε3* homozygotes, *ε4* homozygotes showed a smaller number of spindles with lower power and frequency, which have been robustly linked to neurodegeneration. Thus, altered sleep spindle activity may be one pathway connecting *APOE ε4* homozygosity to AD risk. In addition, *APOE ε4* heterozygotes and homozygotes showed less sleep fragmentation, as shown across multiple measures, including ORP, arousal index, and WASO. Genotype differences in sleep fragmentation became more pronounced with age, such that *APOE ε4* homozygotes showed a monotonic downward trend in sleep fragmentation measures, contrasting with the monotonic upward trend observed among *ε3* homozygotes. The physiological basis of reduced sleep fragmentation in *APOE ε4* carriers remains unclear, but may reflect EEG slowing due to progressive neuronal loss.

## Author Contributions

G.C. and B.M. contributed to the conception and design of the study; G.C., A.C., and B.M. contributed to the acquisition and analysis of data; G.C., E.C., O.B., D.K., A.P.M., and B.M. contributed to drafting the text or preparing the figures.

## Potential Conflicts of Interest

Nothing to report.

## Supporting information


**Figure S1.** Participant flow chart.
**Figure S2.** Kaplan–Meier survival curve by *APOE* genotype.
**Table S0.** Summary of sleep characteristics.
**Table S1.** Association between *APOE* genotype and macro‐level sleep architecture^a^.
**Table S2.** Sex differences in the association between *APOE* genotype and macro‐level sleep architecture^a^.
**Table S3.** Racial differences in the association between *APOE* genotype and macro‐level sleep architecture^a^.
**Table S4.** Differences in the association between *APOE* genotype and macro‐level sleep architecture by cognitive status^a^.
**Table S5.** Differences in the association between *APOE* genotype and macro‐level sleep architecture by pTau181 levels^a^.
**Table S6.** Differences in the association between *APOE* genotype and macro‐level sleep architecture by ab42:40 levels^a^.
**Table S7.** Association between *APOE* genotype and sleep spindle characteristics^a^.
**Table S8.** Association of *APOE* genotype with ORP and arousal index^a^.
**Table S9.** Sex differences in the association between *APOE* genotype and micro‐level sleep architecture^a^.
**Table S10.** Racial differences in the association between *APOE* genotype and micro‐level sleep architecture^a^.
**Table S11.** Differences in the association between *APOE* genotype and micro‐level sleep architecture by cognitive status^a^.
**Table S12.** Differences in the association between *APOE* genotype and micro‐level sleep architecture by pTau181^a^.
**Table S13.** Differences in the association between *APOE* genotype and micro‐level sleep architecture by ab42:40^a^.
**Table S14.** Differences in the association between *APOE* genotype and alternative sleep metrics^a^.
**Table S15.** Association between sleep architecture and *APOE* genotype excluding APOE *ε*2 carriers^a^.
**Table S16.** Sensitivity test of the association between sleep architecture and *APOE* genotype to replacing AHI4% with AHI3%.

## Data Availability

The data used in this study are available through the Biologic Specimen and Data Repository Information Coordinating Center (BioLINCC), the database of Genotypes and Phenotypes (dbGaP), and National Sleep Research Resource (NSRR). Access to these data is subject to approval by the relevant data access committees and is available from respectivedata repositories to qualified researchers upon request.
