# Supplementary material for: An Investigation of Sleep Macro‐ and Microarchitecture by APOE Genotype
Source: Ann Neurol. 2026 Sep 22;100(5):1012–22. doi: 10.1002/ana.78312 (PMC13688065; doi:10.1002/ana.78312)
Supplement: Supplementary file 1 [file ANA-100-1012-s001.docx]

**Supplementary Figure 1.** Participant flow chart

*Acronyms.* ARIC=Atherosclerosis Risk in the Community Study, CHS=Cardiovascular Health Study; FHS=Framingham Heart Study, SHHS=Sleep Heart Health Study

**Supplementary Figure 2.** Kaplan-Meier survival curve by *APOE* genotype

**Supplementary Table 0.** Summary of sleep characteristics

| **Characteristics** | **Mean (standard deviation)** ^a^ | | | | |  |
| --- | --- | --- | --- | --- | --- | --- |
|  | **Full sample**  **(N=3081)** | ***APOE* genotype** | | | |  |
|  |  | ***APOE E*4 heterozygotes**^b^ | ***APOE E*4 homozygotes** | ***APOE* E2 carriers** | ***APOE* E3 homozygotes** | ***p***^c^ |
| **Macro sleep architecture** |  |  |  |  |  |  |
| **%REM** | 19.9 (6.14) | 19.8 (6.37) | 20.2 (6.50) | 19.9 (5.86) | 20.0 (6.09) | 0.96 |
| **%N1** | 5.4 (3.97) | 5.4 (4.13) | 5.7 (3.71) | 5.7 (4.17) | 5.4 (3.85) | 0.56 |
| **%N2** | 56.1 (11.85) | 55.9 (11.59) | 54.2 (11.88) | 56.2 (12.20) | 56.1 (11.88) | 0.58 |
| **%N3** | 18.6 (12.12) | 18.8 (11.94) | 19.9 (11.14) | 18.2 (12.34) | 18.5 (12.18) | 0.65 |
| **WASO** | 64.5 (44.36) | 64.1 (47.61) | 61.7 (49.50) | 64.4 (41.69) | 64.8 (43.36) | 0.94 |
| **Micro sleep architecture** |  |  |  |  |  |  |
| **Spindle power** | 28.13 (9.64) | 28.71 (9.91) | 26.53 (9.02) | 28.04 (9.61) | 27.97 (9.55) | 0.16 |
| **Spindle density** | 1.05 (0.71) | 1.06 (0.73) | 0.95 (0.67) | 1.08 (0.76) | 1.04 (0.69) | 0.50 |
| **Spindle frequency** | 11.93 (0.55) | 11.91 (0.54) | 11.84 (0.56) | 11.98 (0.55) | 11.93 (0.56) | 0.10 |
| **Total sleep ORP** | 0.93 (0.23) | 0.91 (0.23) | 0.87 (0.21) | 0.95 (0.23) | 0.94 (0.23) | <0.01 |
| **Arousal index** | 19.5 (10.50) | 19.1 (10.51) | 18.0 (8.14) | 19.1 (9.79) | 19.9 (10.71) | 0.13 |

Acronyms. APOE= apolipoprotein E; IQR=interquartile range; REMS=rapid eye movement; SWS=slow wave sleep; WASO=wake after sleep onset

**Supplementary Table 1.** Association between *APOE* genotype and macro-level sleep architecture ^a^

| **Model** | **Sleep Characteristics** | ***APOE* genotype** ^b^ | | | | | | | |
| --- | --- | --- | --- | --- | --- | --- | --- | --- | --- |
|  |  | ***APOE* ε4 Heterozygote** | |  | ***APOE* ε4 Homozygote** | |  | ***APOE* ε2 carriers** | |
|  |  | **β** | ***p*** |  | **β** | ***p*** |  | **β** | ***p*** |
| **No interaction** | **%REM** | -0.074 | 0.778 |  | 0.197 | 0.810 |  | 0.033 | 0.916 |
|  | **%N1** | -0.003 | 0.987 |  | 0.271 | 0.553 |  | 0.328 | 0.127 |
|  | **%N2** | -0.075 | 0.872 |  | -1.452 | 0.312 |  | 0.143 | 0.820 |
|  | **%N3** | 0.151 | 0.744 |  | 0.985 | 0.441 |  | -0.503 | 0.418 |
|  | **WASO** | -1.149 | 0.512 |  | -4.142 | 0.473 |  | -1.108 | 0.580 |
| **Age x *APOE*** | **Estimate at mean age** | **β** | ***p*** |  | **β** | ***p*** |  | **β** | ***p*** |
|  | **%REM** | -0.078 | 0.766 |  | 0.197 | 0.816 |  | 0.039 | 0.901 |
|  | **%N1** | -0.002 | 0.990 |  | 0.233 | 0.617 |  | 0.326 | 0.128 |
|  | **%N2** | -0.075 | 0.873 |  | -1.330 | 0.364 |  | 0.132 | 0.832 |
|  | **%N3** | 0.154 | 0.739 |  | 0.901 | 0.482 |  | -0.497 | 0.424 |
|  | **WASO** | -1.129 | 0.521 |  | -5.099 | 0.363 |  | -1.112 | 0.578 |
|  | **Differences by 1 year of age** | **β** | ***p*** |  | **β** | ***p*** |  | **β** | ***p*** |
|  | **%REM** | -0.049 | 0.104 |  | -0.023 | 0.829 |  | -0.075 | 0.028* |
|  | **%N1** | 0.008 | 0.648 |  | -0.062 | 0.252 |  | 0.015 | 0.520 |
|  | **%N2** | -0.008 | 0.872 |  | 0.222 | 0.236 |  | 0.120 | 0.087 |
|  | **%N3** | 0.049 | 0.354 |  | -0.137 | 0.381 |  | -0.059 | 0.432 |
|  | **WASO** | 0.303 | 0.126 |  | -1.568 | 0.025* |  | 0.094 | 0.663 |

^a^ Covariates include age, sex, race, marital status, educational attainment, parent cohort, probable dementia, hypertension, diabetes, body mass index, total sleep time, apnea-hypopnea index, and the frequency of sleeping pill intake.

^b^ Reference group is *APOE* ε3 homozygotes.

* Denotes statistical significance at the level of *p*<.0.05.

Acronyms. APOE=apolipoprotein E; AHI4%=apnea-hypopnea index using the 4% desaturation criterion; REM=rapid eye movement

**Supplementary Table 2.** Sex differences in the association between *APOE* genotype and macro-level sleep architecture ^a^

| **Sleep Characteristics** | ***APOE* genotype** ^b^ | | | | | | | |
| --- | --- | --- | --- | --- | --- | --- | --- | --- |
|  | ***APOE* ε4 Heterozygote** | |  | ***APOE* ε4 Homozygote** | |  | ***APOE* ε2 carriers** | |
|  | **β** | ***p*** |  | **β** | ***p*** |  | **β** | ***p*** |
| **Men** |  |  |  |  |  |  |  |  |
| **%REM** | 0.062 | 0.871 |  | 2.055 | 0.127 |  | 0.035 | 0.940 |
| **%N1** | 0.068 | 0.812 |  | 0.058 | 0.941 |  | 0.492 | 0.196 |
| **%N2** | -0.939 | 0.144 |  | -3.817 | 0.098 |  | -1.349 | 0.114 |
| **%N3** | 0.810 | 0.203 |  | 1.705 | 0.414 |  | 0.822 | 0.351 |
| **WASO** | -3.457 | 0.216 |  | 2.912 | 0.775 |  | -0.122 | 0.969 |
| **Differences in women** | **β** | ***p*** |  | **β** | ***p*** |  | **β** | ***p*** |
| **%REM** | -0.256 | 0.623 |  | -3.327 | 0.046* |  | -0.014 | 0.982 |
| **%N1** | -0.133 | 0.691 |  | 0.375 | 0.694 |  | -0.303 | 0.497 |
| **%N2** | 1.636 | 0.074 |  | 4.296 | 0.143 |  | 2.763 | 0.026* |
| **%N3** | -1.247 | 0.173 |  | -1.344 | 0.610 |  | -2.446 | 0.048* |
| **WASO** | 4.288 | 0.227 |  | -12.560 | 0.301 |  | -1.758 | 0.665 |

^a^ Estimates are derived from models including sex-by-genotype interactions, as well as age, sex, race, marital status, educational attainment, parent cohort, probable dementia, hypertension, diabetes, body mass index, total sleep time, apnea-hypopnea index, and the frequency of sleeping pill intake.

^b^ Reference group is *APOE* ε3 homozygotes.

* Denotes statistical significance at the level of *p*<.0.05.

*Acronyms.* APOE=apolipoprotein E; AHI4%=apnea-hypopnea index using the 4% desaturation criterion; REM=rapid eye movement

**Supplementary Table 3.** Racial differences in the association between *APOE* genotype and macro-level sleep architecture ^a^

| **Sleep Characteristics** | ***APOE* genotype** ^b^ | | | | | |
| --- | --- | --- | --- | --- | --- | --- |
|  | ***APOE* ε4 carriers** | |  | ***APOE* ε2 carriers** | |  |
|  | **β** | ***p*** |  | **β** | ***p*** |  |
| **White** |  |  |  |  |  |  |
| **%REM** | -0.076 | 0.767 |  | 0.080 | 0.804 |  |
| **%N1** | 0.045 | 0.774 |  | 0.452 | 0.044* |  |
| **%N2** | -0.182 | 0.693 |  | 0.048 | 0.940 |  |
| **%N3** | 0.213 | 0.644 |  | -0.580 | 0.371 |  |
| **WASO** | -1.240 | 0.472 |  | -0.663 | 0.747 |  |
| **Differences in Black** | **β** | ***p*** |  | **β** | ***p*** |  |
| **%REM** | 0.439 | 0.767 |  | -0.538 | 0.678 |  |
| **%N1** | -0.599 | 0.573 |  | -1.701 | 0.027* |  |
| **%N2** | 0.045 | 0.985 |  | 1.235 | 0.627 |  |
| **%N3** | 0.116 | 0.955 |  | 1.004 | 0.666 |  |
| **WASO** | -3.126 | 0.753 |  | -6.223 | 0.476 |  |

^a^ Estimates are derived from models including race-by-genotype interactions, as well as age, sex, race, marital status, educational attainment, parent cohort, probable dementia, hypertension, diabetes, body mass index, total sleep time, apnea-hypopnea index, and the frequency of sleeping pill intake.

^b^ Reference group is *APOE* ε3 homozygotes.

* Denotes statistical significance at the level of *p*<.0.05.

*Acronyms.* APOE=apolipoprotein E; AHI4%=apnea-hypopnea index using the 4% desaturation criterion; REM=rapid eye movement

**Supplementary Table 4.** Differences in the association between *APOE* genotype and macro-level sleep architecture by cognitive status ^a^

| **Sleep Characteristics** | ***APOE* genotype** ^b^ | | | | | |  |
| --- | --- | --- | --- | --- | --- | --- | --- |
|  | ***APOE* ε4 carriers** | |  | ***APOE* ε2 carriers** | |  | |
|  | **β** | ***p*** |  | **β** | ***p*** |  | |
| **No dementia** |  |  |  |  |  |  | |
| **%REM** | -0.054 | 0.834 |  | 0.074 | 0.813 |  | |
| **%N1** | 0.018 | 0.912 |  | 0.239 | 0.247 |  | |
| **%N2** | -0.165 | 0.719 |  | 0.244 | 0.700 |  | |
| **%N3** | 0.201 | 0.656 |  | -0.557 | 0.375 |  | |
| **WASO** | -1.242 | 0.470 |  | -1.161 | 0.563 |  | |
| **Differences in probable dementia** | **β** | ***p*** |  | **β** | ***p*** |  | |
| **%REM** | -0.115 | 0.948 |  | -2.427 | 0.233 |  | |
| **%N1** | 0.381 | 0.750 |  | 5.179 | 0.218 |  | |
| **%N2** | -1.024 | 0.738 |  | -5.882 | 0.200 |  | |
| **%N3** | 0.758 | 0.819 |  | 3.130 | 0.476 |  | |
| **WASO** | -4.984 | 0.721 |  | 3.202 | 0.894 |  | |

^a^ Estimates are derived from models including cognitive status-by-genotype interactions, as well as age, sex, race, marital status, educational attainment, parent cohort, probable dementia, hypertension, diabetes, body mass index, total sleep time, apnea-hypopnea index, and the frequency of sleeping pill intake.

^b^ Reference group is *APOE* ε3 homozygotes.

* Denotes statistical significance at the level of *p*<.0.05.

*Acronyms.* APOE=apolipoprotein E; AHI4%=apnea-hypopnea index using the 4% desaturation criterion; REM=rapid eye movement

**Supplementary Table 5.** Differences in the association between *APOE* genotype and macro-level sleep architecture by pTau181 levels ^a^

| **Sleep Characteristics** | ***APOE* genotype** ^b^ | | | | | |
| --- | --- | --- | --- | --- | --- | --- |
|  | ***APOE* ε4 carriers** | |  | ***APOE* ε2 carriers** | |  |
|  | **β** | ***p*** |  | **β** | ***p*** |  |
| **Estimates at mean ptau181** ^c^ |  |  |  |  |  |  |
| **%REM** | -0.343 | 0.609 |  | 0.281 | 0.726 |  |
| **%N1** | -0.484 | 0.247 |  | 0.018 | 0.972 |  |
| **%N2** | -1.298 | 0.278 |  | -0.103 | 0.945 |  |
| **%N3** | 2.125 | 0.068 |  | -0.195 | 0.882 |  |
| **WASO** | -1.029 | 0.813 |  | 2.068 | 0.658 |  |
| **Differences by 1 unit of ptau181** | **β** | ***p*** |  | **β** | ***p*** |  |
| **%REM** | -0.523 | 0.200 |  | -0.809 | 0.137 |  |
| **%N1** | 0.372 | 0.190 |  | 0.531 | 0.184 |  |
| **%N2** | 0.000 | 1.000 |  | 0.713 | 0.537 |  |
| **%N3** | 0.150 | 0.836 |  | -0.436 | 0.642 |  |
| **WASO** | -3.177 | 0.295 |  | -1.343 | 0.624 |  |

^a^ Estimates are derived from models including biomarker-by-genotype interactions, as well as age, sex, race, marital status, educational attainment, parent cohort, hypertension, diabetes, body mass index, total sleep time, apnea-hypopnea index, and the frequency of sleeping pill intake.

^b^ Reference group is *APOE* ε3 homozygotes.

^c^ Mean was calculated among non-dementia individuals only, so that the model estimates are interpreted relative to a biomarker level typical of individuals without dementia.

* Denotes statistical significance at the level of *p*<.0.05.

*Acronyms.* APOE=apolipoprotein E; AHI4%=apnea-hypopnea index using the 4% desaturation criterion; REM=rapid eye movement

**Supplementary Table 6.** Differences in the association between *APOE* genotype and macro-level sleep architecture by ab42:40 levels ^a^

| **Sleep Characteristics** | ***APOE* genotype** ^b^ | | | | | |
| --- | --- | --- | --- | --- | --- | --- |
|  | ***APOE* ε4 carriers** | |  | ***APOE* ε2 carriers** | |  |
|  | **β** | ***p*** |  | **β** | ***p*** |  |
| **Estimates at mean ab42:40** ^c^ |  |  |  |  |  |  |
| **%REM** | 0.257 | 0.580 |  | 0.636 | 0.243 |  |
| **%N1** | -0.237 | 0.425 |  | 0.013 | 0.970 |  |
| **%N2** | -1.320 | 0.111 |  | -1.173 | 0.271 |  |
| **%N3** | 1.299 | 0.104 |  | 0.524 | 0.586 |  |
| **WASO** | -0.134 | 0.966 |  | -2.540 | 0.434 |  |
| **Differences by 1 unit of ab42:40** | **β** | ***p*** |  | **β** | ***p*** |  |
| **%REM** | 2.522 | 0.483 |  | 1.310 | 0.764 |  |
| **%N1** | 0.704 | 0.731 |  | -1.239 | 0.515 |  |
| **%N2** | 10.570 | 0.105 |  | -0.040 | 0.996 |  |
| **%N3** | -13.796 | 0.030^*, d^ |  | -0.031 | 0.996 |  |
| **WASO** | 37.020 | 0.148 |  | -12.589 | 0.650 |  |

^a^ Estimates are derived from models including biomarker-by-genotype interactions, as well as age, sex, race, marital status, educational attainment, parent cohort, hypertension, diabetes, body mass index, total sleep time, apnea-hypopnea index, and the frequency of sleeping pill intake.

^b^ Reference group is *APOE* ε3 homozygotes.

^c^ Mean was calculated among non-dementia individuals only, so that the model estimates are interpreted relative to a biomarker level typical of individuals without dementia.

^d^ Given differences in the timing of blood sample collection between the two cohorts included in this study, additional analyses were conducted separately within each cohort. The interaction between APOE genotype and the Aβ42:40 ratio was no longer significantly associated with %N3 in either cohort, although the β estimates remained comparable in magnitude and direction to those reported here.

* Denotes statistical significance at the level of *p*<.0.05.

*Acronyms.* APOE=apolipoprotein E; AHI4%=apnea-hypopnea index using the 4% desaturation criterion; REM=rapid eye movement

**Supplementary Table 7.** Association between *APOE* genotype and sleep spindle characteristics ^a^

| **Model** | **Spindle characteristics** | ***APOE* genotype** ^b^ | | | | | | | |
| --- | --- | --- | --- | --- | --- | --- | --- | --- | --- |
|  |  | ***APOE* ε4 Heterozygote** | |  | ***APOE* ε4 Homozygote** | |  | ***APOE* ε2 carriers** | |
|  |  | **β** | ***p*** |  | **β** | ***p*** |  | **β** | ***p*** |
| **No interaction** | **Power** | 0.342 | 0.367 |  | -2.135 | 0.024* |  | -0.081 | 0.860 |
|  | **Density** | -0.007 | 0.803 |  | -0.138 | 0.057 |  | 0.034 | 0.351 |
|  | **Frequency** | -0.044 | 0.041* |  | -0.116 | 0.065 |  | 0.038 | 0.174 |
| **Age x *APOE*** | **Estimate at mean age** | **β** | ***p*** |  | **β** | ***p*** |  | **β** | ***p*** |
|  | **Power** | 0.343 | 0.364 |  | -2.172 | 0.026* |  | -0.080 | 0.862 |
|  | **Density** | -0.007 | 0.796 |  | -0.141 | 0.052 |  | 0.034 | 0.355 |
|  | **Frequency** | -0.044 | 0.042* |  | -0.122 | 0.049* |  | 0.038 | 0.175 |
|  | **Differences by 1 year of age** | **β** | ***p*** |  | **β** | ***p*** |  | **β** | ***p*** |
|  | **Power** | 0.022 | 0.617 |  | -0.058 | 0.657 |  | -0.006 | 0.900 |
|  | **Density** | -0.003 | 0.383 |  | -0.006 | 0.506 |  | 0.001 | 0.766 |
|  | **Frequency** | 0.001 | 0.719 |  | -0.010 | 0.168 |  | 0.001 | 0.770 |

^a^ Covariates include age, sex, race, marital status, educational attainment, parent cohort, probable dementia, hypertension, diabetes, body mass index, total sleep time, apnea-hypopnea index, and the frequency of sleeping pill intake.

^b^ Reference group is *APOE* ε3 homozygotes.

* Denotes statistical significance at the level of *p*<.0.05.

*Acronyms*. APOE=apolipoprotein E; AHI4%=apnea-hypopnea index using the 4% desaturation criterion; REM=rapid eye movement

**Supplementary Table 8.** Association of *APOE* genotype with ORP and arousal index ^a^

| **Model** | **Sleep characteristic** | ***APOE* genotype** ^b^ | | | | | | | |
| --- | --- | --- | --- | --- | --- | --- | --- | --- | --- |
|  |  | ***APOE* ε4 Heterozygote** | |  | ***APOE* ε4 Homozygote** | |  | ***APOE* ε2 carriers** | |
|  |  | **β** | ***p*** |  | **β** | ***p*** |  | **β** | ***p*** |
| **No interaction** | **ORP average by sleep period** |  |  |  |  |  |  |  |  |
|  | **Total sleep** | -0.031 | 0.002* |  | -0.069 | 0.008* |  | 0.004 | 0.724 |
|  | **N1 average** | -0.025 | 0.038* |  | -0.058 | 0.071 |  | 0.002 | 0.891 |
|  | **N2 average** | -0.030 | 0.005* |  | -0.067 | 0.017* |  | -0.001 | 0.927 |
|  | **N3 average** | -0.023 | 0.002* |  | -0.046 | 0.012* |  | -0.003 | 0.780 |
|  | **REM average** | -0.036 | 0.012* |  | -0.070 | 0.111 |  | 0.004 | 0.821 |
|  | **Arousal Index** | -1.021 | 0.006* |  | -1.781 | 0.074 |  | -0.893 | 0.058 |
| **Age x *APOE*** | **Estimate at mean age** | **β** | ***p*** |  | **β** | ***p*** |  | **β** | ***p*** |
|  | **ORP average by sleep period** |  |  |  |  |  |  |  |  |
|  | **Total sleep** | -0.031 | 0.002* |  | -0.072 | 0.005* |  | 0.004 | 0.727 |
|  | **N1 average** | -0.025 | 0.036* |  | -0.061 | 0.067 |  | 0.002 | 0.902 |
|  | **N2 average** | -0.030 | 0.004* |  | -0.071 | 0.010* |  | -0.001 | 0.924 |
|  | **N3 average** | -0.023 | 0.002* |  | -0.047 | 0.013* |  | -0.003 | 0.782 |
|  | **REM average** | -0.036 | 0.011* |  | -0.075 | 0.093 |  | 0.004 | 0.825 |
|  | **Arousal Index** | -1.023 | 0.006* |  | -1.912 | 0.049* |  | -0.899 | 0.056 |
|  | **Differences by 1 year of age** | **β** | ***p*** |  | **β** | ***p*** |  | **β** | ***p*** |
|  | **ORP average by sleep period** |  |  |  |  |  |  |  |  |
|  | **Total sleep** | -0.003 | 0.013* |  | -0.007 | 0.037* |  | 0.000 | 0.989 |
|  | **N1 average** | -0.003 | 0.037* |  | -0.005 | 0.258 |  | 0.002 | 0.284 |
|  | **N2 average** | -0.002 | 0.069 |  | -0.008 | 0.022* |  | 0.000 | 0.922 |
|  | **N3 average** | -0.001 | 0.474 |  | -0.003 | 0.379 |  | -0.001 | 0.465 |
|  | **REM average** | -0.004 | 0.017* |  | -0.010 | 0.083 |  | 0.001 | 0.807 |
|  | **Arousal Index** | -0.037 | 0.401 |  | -0.237 | 0.064 |  | 0.060 | 0.261 |

^a^ Covariates include age, sex, race, marital status, educational attainment, parent cohort, probable dementia, hypertension, diabetes, body mass index, total sleep time, apnea-hypopnea index, and the frequency of sleeping pill intake.

^b^ Reference group is *APOE* ε3 homozygotes.

* Denotes statistical significance at the level of *p*<.0.05.

*Acronyms.* APOE=apolipoprotein E; AHI4%=apnea-hypopnea index using the 4% desaturation criterion; ORP=odds ratio product

**Supplementary Table 9.** Sex differences in the association between *APOE* genotype and micro-level sleep architecture ^a^

| **Sleep Characteristics** | ***APOE* genotype** ^b^ | | | | | | | |
| --- | --- | --- | --- | --- | --- | --- | --- | --- |
|  | ***APOE* ε4 Heterozygote** | |  | ***APOE* ε4 Homozygote** | |  | ***APOE* ε2 carriers** | |
|  | **β** | ***p*** |  | **β** | ***p*** |  | **β** | ***p*** |
| **Men** |  |  |  |  |  |  |  |  |
| **Spindle Power** | 0.334 | 0.495 |  | -3.591 | 0.004* |  | -0.233 | 0.711 |
| **Spindle Density** | -0.035 | 0.330 |  | -0.189 | 0.067 |  | 0.010 | 0.841 |
| **Spindle Frequency** | -0.055 | 0.086 |  | -0.120 | 0.251 |  | 0.031 | 0.457 |
| **Total sleep average** | -0.030 | 0.026* |  | -0.100 | 0.013* |  | -0.009 | 0.593 |
| **N1 average ORP** | -0.023 | 0.170 |  | -0.097 | 0.073 |  | -0.011 | 0.625 |
| **N2 average ORP** | -0.028 | 0.046* |  | -0.108 | 0.010* |  | -0.008 | 0.676 |
| **N3 average ORP** | -0.024 | 0.025* |  | -0.049 | 0.110 |  | -0.009 | 0.512 |
| **REM average ORP** | -0.030 | 0.123 |  | -0.106 | 0.109 |  | -0.021 | 0.386 |
| **Arousal Index** | -0.970 | 0.095 |  | -2.639 | 0.012* |  | -1.914 | 0.009* |
| **Differences in women** | **β** | ***p*** |  | **β** | ***p*** |  | **β** | ***p*** |
| **Spindle power** | 0.020 | 0.979 |  | 2.607 | 0.161 |  | 0.282 | 0.759 |
| **Spindle density** | 0.052 | 0.329 |  | 0.094 | 0.519 |  | 0.045 | 0.537 |
| **Spindle frequency** | 0.020 | 0.643 |  | 0.007 | 0.957 |  | 0.013 | 0.813 |
| **Total sleep average** | -0.001 | 0.974 |  | 0.056 | 0.287 |  | 0.025 | 0.305 |
| **N1 average ORP** | -0.003 | 0.901 |  | 0.070 | 0.293 |  | 0.024 | 0.432 |
| **N2 average ORP** | -0.002 | 0.912 |  | 0.074 | 0.187 |  | 0.012 | 0.647 |
| **N3 average ORP** | 0.002 | 0.896 |  | 0.004 | 0.917 |  | 0.011 | 0.563 |
| **REM average ORP** | -0.011 | 0.712 |  | 0.065 | 0.468 |  | 0.046 | 0.192 |
| **Arousal Index** | -0.081 | 0.914 |  | 1.559 | 0.411 |  | 1.870 | 0.051 |

^a^ Estimates are derived from models including sex-by-genotype interactions, as well as age, sex, race, marital status, educational attainment, parent cohort, probable dementia, hypertension, diabetes, body mass index, total sleep time, apnea-hypopnea index, and the frequency of sleeping pill intake.

^b^ Reference group is *APOE* ε3 homozygotes.

* Denotes statistical significance at the level of *p*<.0.05.

*Acronyms.* APOE=apolipoprotein E; AHI4%=apnea-hypopnea index using the 4% desaturation criterion; ORP=odds ratio product;

REM=rapid eye movement

**Supplementary Table 10.** Racial differences in the association between *APOE* genotype and micro-level sleep architecture ^a^

| **Sleep Characteristics** | ***APOE* genotype** ^b^ | | | | | |
| --- | --- | --- | --- | --- | --- | --- |
|  | ***APOE* ε4 carriers** | |  | ***APOE* ε2 carriers** | |  |
|  | **β** | ***p*** |  | **β** | ***p*** |  |
| **White** |  |  |  |  |  |  |
| **Spindle power** | 0.192 | 0.612 |  | -0.134 | 0.781 |  |
| **Spindle density** | -0.014 | 0.623 |  | 0.033 | 0.398 |  |
| **Spindle frequency** | -0.048 | 0.028* |  | 0.039 | 0.173 |  |
| **Total sleep average ORP** | -0.029 | 0.003* |  | 0.005 | 0.719 |  |
| **N1 average ORP** | -0.024 | 0.039* |  | -0.004 | 0.811 |  |
| **N2 average ORP** | -0.028 | 0.006* |  | -0.003 | 0.825 |  |
| **N3 average ORP** | -0.023 | 0.001* |  | -0.003 | 0.730 |  |
| **REM average ORP** | -0.034 | 0.017* |  | 0.004 | 0.829 |  |
| **Arousal Index** | -1.121 | 0.002* |  | -0.718 | 0.136 |  |
| **Differences in Black** | **β** | ***p*** |  | **β** | ***p*** |  |
| **Spindle power** | -0.850 | 0.545 |  | 0.557 | 0.747 |  |
| **Spindle density** | -0.071 | 0.458 |  | 0.008 | 0.945 |  |
| **Spindle frequency** | -0.040 | 0.655 |  | -0.024 | 0.829 |  |
| **Total sleep average ORP** | -0.091 | 0.053 |  | -0.017 | 0.727 |  |
| **N1 average ORP** | -0.058 | 0.305 |  | 0.067 | 0.265 |  |
| **N2 average ORP** | -0.085 | 0.090 |  | 0.010 | 0.849 |  |
| **N3 average ORP** | -0.028 | 0.491 |  | 0.005 | 0.905 |  |
| **REM average ORP** | -0.089 | 0.165 |  | -0.013 | 0.843 |  |
| **Arousal Index** | 0.704 | 0.692 |  | -2.153 | 0.319 |  |

^a^ Estimates are derived from models including race-by-genotype interactions, as well as age, sex, race, marital status, educational attainment, parent cohort, probable dementia, hypertension, diabetes, body mass index, total sleep time, apnea-hypopnea index, and the frequency of sleeping pill intake.

^b^ Reference group is *APOE* ε3 homozygotes.

* Denotes statistical significance at the level of *p*<.0.05.

*Acronyms.* APOE=apolipoprotein E; AHI4%=apnea-hypopnea index using the 4% desaturation criterion; ORP=odds ratio product; REM=rapid eye movement

**Supplementary Table 11.** Differences in the association between *APOE* genotype and micro-level sleep architecture by cognitive status ^a^

| **Sleep Characteristics** | ***APOE* genotype** ^b^ | | | | | |
| --- | --- | --- | --- | --- | --- | --- |
|  | ***APOE* ε4 carriers** | |  | ***APOE* ε2 carriers** | |  |
|  | **β** | ***p*** |  | **β** | ***p*** |  |
| **No dementia** |  |  |  |  |  |  |
| **Spindle power** | 0.248 | 0.504 |  | -0.037 | 0.938 |  |
| **Spindle density** | -0.012 | 0.651 |  | 0.039 | 0.292 |  |
| **Spindle frequency** | -0.051 | 0.016* |  | 0.043 | 0.124 |  |
| **Total sleep average ORP** | -0.030 | 0.002* |  | 0.007 | 0.583 |  |
| **N1 average ORP** | -0.024 | 0.037* |  | 0.004 | 0.809 |  |
| **N2 average ORP** | -0.029 | 0.004* |  | 0.001 | 0.924 |  |
| **N3 average ORP** | -0.024 | 0.001* |  | -0.001 | 0.946 |  |
| **REM average ORP** | -0.034 | 0.016* |  | 0.009 | 0.634 |  |
| **Arousal Index** | -0.974 | 0.007* |  | -0.889 | 0.062 |  |
| **Differences in probable dementia** | **β** | ***p*** |  | **β** | ***p*** |  |
| **Spindle power** | -3.824 | 0.083 |  | -2.525 | 0.178 |  |
| **Spindle density** | -0.199 | 0.148 |  | -0.292 | 0.028* |  |
| **Spindle frequency** | 0.043 | 0.767 |  | -0.306 | 0.198 |  |
| **Total sleep average ORP** | -0.123 | 0.052 |  | -0.145 | 0.030* |  |
| **N1 average ORP** | -0.121 | 0.175 |  | -0.093 | 0.233 |  |
| **N2 average ORP** | -0.130 | 0.053 |  | -0.140 | 0.026* |  |
| **N3 average ORP** | -0.020 | 0.701 |  | -0.113 | 0.038* |  |
| **REM average ORP** | -0.187 | 0.056 |  | -0.265 | 0.014* |  |
| **Arousal Index** | -3.893 | 0.151 |  | -0.190 | 0.945 |  |

^a^ Estimates are derived from models including cognitive status-by-genotype interactions, as well as age, sex, race, marital status, educational attainment, parent cohort, probable dementia, hypertension, diabetes, body mass index, total sleep time, apnea-hypopnea index, and the frequency of sleeping pill intake.

^b^ Reference group is *APOE* ε3 homozygotes.

* Denotes statistical significance at the level of *p*<.0.05.

*Acronyms.* APOE=apolipoprotein; AHI4%=apnea-hypopnea index using the 4% desaturation criterion; ORP=odds ratio product; REM=rapid eye movement

**Supplementary Table 12.** Differences in the association between *APOE* genotype and micro-level sleep architecture by pTau181 ^a^

| **Sleep Characteristics** | ***APOE* genotype** ^b^ | | | | | |
| --- | --- | --- | --- | --- | --- | --- |
|  | ***APOE* ε4 carriers** | |  | ***APOE* ε2 carriers** | |  |
|  | **β** | ***p*** |  | **β** | ***p*** |  |
| **Estimates at mean ptau181** ^c^ |  |  |  |  |  |  |
| **Spindle power** | 1.441 | 0.074 |  | 0.282 | 0.762 |  |
| **Spindle density** | -0.004 | 0.932 |  | 0.067 | 0.369 |  |
| **Spindle frequency** | -0.035 | 0.439 |  | 0.109 | 0.095 |  |
| **Total sleep average ORP** | -0.075 | 0.001* |  | -0.017 | 0.590 |  |
| **N1 average ORP** | -0.058 | 0.045* |  | -0.007 | 0.873 |  |
| **N2 average ORP** | -0.061 | 0.010* |  | -0.020 | 0.535 |  |
| **N3 average ORP** | -0.033 | 0.055 |  | -0.022 | 0.301 |  |
| **REM average ORP** | -0.064 | 0.051 |  | -0.016 | 0.704 |  |
| **Arousal Index** | -2.263 | 0.018* |  | -1.486 | 0.202 |  |
| **Differences by 1 unit of ptau181** | **β** | ***p*** |  | **β** | ***p*** |  |
| **Spindle power** | 0.064 | 0.918 |  | -0.498 | 0.468 |  |
| **Spindle density** | 0.012 | 0.740 |  | 0.006 | 0.905 |  |
| **Spindle frequency** | -0.011 | 0.754 |  | 0.029 | 0.563 |  |
| **Total sleep average ORP** | -0.019 | 0.234 |  | -0.031 | 0.163 |  |
| **N1 average ORP** | -0.020 | 0.244 |  | -0.048 | 0.165 |  |
| **N2 average ORP** | -0.012 | 0.480 |  | -0.026 | 0.238 |  |
| **N3 average ORP** | -0.003 | 0.818 |  | -0.020 | 0.153 |  |
| **REM average ORP** | -0.042 | 0.095 |  | -0.051 | 0.093 |  |
| **Arousal Index** | -0.223 | 0.688 |  | -0.660 | 0.394 |  |

^a^ Estimates are derived from models including biomarker-by-genotype interactions, as well as age, sex, race, marital status, educational attainment, parent cohort, hypertension, diabetes, body mass index, total sleep time, apnea-hypopnea index, and the frequency of sleeping pill intake.

^b^ Reference group is *APOE* ε3 homozygotes.

^c^ Mean was calculated among non-dementia individuals only, so that the model estimates are interpreted relative to a biomarker level typical of individuals without dementia.

* Denotes statistical significance at the level of *p*<.0.05.

*Acronyms.* APOE=apolipoprotein E; AHI4%=apnea-hypopnea index using the 4% desaturation criterion; ORP=odds ratio product; REM=rapid eye movement

**Supplementary Table 13.** Differences in the association between *APOE* genotype and micro-level sleep architecture by ab42:40 ^a^

| **Sleep Characteristics** | ***APOE* genotype** ^b^ | | | | | |
| --- | --- | --- | --- | --- | --- | --- |
|  | ***APOE* ε4 carriers** | |  | ***APOE* ε2 carriers** | |  |
|  | **β** | ***p*** |  | **β** | ***p*** |  |
| **Estimates at mean ab42:40** ^c^ |  |  |  |  |  |  |
| **Spindle power** | 1.313 | 0.037* |  | -0.756 | 0.283 |  |
| **Spindle density** | 0.064 | 0.145 |  | 0.006 | 0.920 |  |
| **Spindle frequency** | -0.043 | 0.217 |  | 0.027 | 0.558 |  |
| **Total sleep average ORP** | -0.062 | 0.000* |  | -0.035 | 0.094 |  |
| **N1 average ORP** | -0.044 | 0.034* |  | -0.030 | 0.253 |  |
| **N2 average ORP** | -0.057 | 0.002* |  | -0.040 | 0.066 |  |
| **N3 average ORP** | -0.034 | 0.007* |  | -0.019 | 0.194 |  |
| **REM average ORP** | -0.072 | 0.004* |  | -0.050 | 0.095 |  |
| **Arousal Index** | -2.099 | 0.001* |  | -1.511 | 0.047* |  |
| **Differences by 1 unit of ab42:40** | **β** | ***p*** |  | **β** | ***p*** |  |
| **Spindle power** | -0.831 | 0.861 |  | -5.436 | 0.220 |  |
| **Spindle density** | 0.318 | 0.343 |  | -0.311 | 0.391 |  |
| **Spindle frequency** | 0.067 | 0.820 |  | -0.273 | 0.292 |  |
| **Total sleep average ORP** | 0.224 | 0.084 |  | -0.100 | 0.479 |  |
| **N1 average ORP** | 0.164 | 0.292 |  | -0.230 | 0.196 |  |
| **N2 average ORP** | 0.100 | 0.450 |  | -0.094 | 0.515 |  |
| **N3 average ORP** | 0.069 | 0.439 |  | 0.067 | 0.543 |  |
| **REM average ORP** | 0.276 | 0.158 |  | -0.204 | 0.332 |  |
| **Arousal Index** | 3.946 | 0.398 |  | 2.753 | 0.623 |  |

^a^ Estimates are derived from models including biomarker-by-genotype interactions, as well as age, sex, race, marital status, educational attainment, parent cohort, hypertension, diabetes, body mass index, total sleep time, apnea-hypopnea index, and the frequency of sleeping pill intake.

^b^ Reference group is *APOE* ε3 homozygotes.

^c^ Mean was calculated among non-dementia individuals only, so that the model estimates are interpreted relative to a biomarker level typical of individuals without dementia.

* Denotes statistical significance at the level of *p*<.0.05.

*Acronyms.* APOE=apolipoprotein E; AHI4%=apnea-hypopnea index using the 4% desaturation criterion; ORP=odds ratio product; REM=rapid eye movement

**Supplementary Table 14.** Differences in the association between *APOE* genotype and alternative sleep metrics ^a^

| **Sleep Characteristics** | ***APOE* genotype** ^b^ | | | | | | | |
| --- | --- | --- | --- | --- | --- | --- | --- | --- |
|  | ***APOE* ε4 Heterozygote** | |  | ***APOE* ε4 Homozygote** | |  | ***APOE* ε2 carriers** | |
|  | **β** | ***p*** |  | **β** | ***p*** |  | **β** | ***p*** |
| **Total Sleep Time** | -1.573 | 0.561 |  | -3.107 | 0.695 |  | -1.386 | 0.670 |
| **AHI4%** | 1.296 | 0.016* |  | 0.183 | 0.899 |  | 0.622 | 0.346 |
| **Epworth Sleepiness Scale**^c^ | -0.027 | 0.883 |  | -0.850 | 0.074 |  | 0.127 | 0.590 |

^a^ Covariates include age, sex, race, marital status, educational attainment, parent cohort, probable dementia, hypertension, diabetes, body mass index, total sleep time, apnea-hypopnea index, and the frequency of sleeping pill intake. For models examining total sleep time and AHI4%, the respective variables were not included as covariates.

^b^ Reference group is *APOE* ε3 homozygotes.

^c^ 80 individuals were missing Epworth Sleepiness Scale scores.

* Denotes statistical significance at the level of *p*<.0.05.

*Acronyms.* AHI4%=apnea-hypopnea index using the 4% desaturation criterion; APOE=apolipoprotein E

**Supplementary Table 15.** Association between sleep architecture and *APOE* genotype excluding APOE ε2 carriers ^a^

| **Sleep Characteristics** |  | ***APOE* ε4 Heterozygote** | |  | ***APOE* ε4 Homozygote** | |
| --- | --- | --- | --- | --- | --- | --- |
|  |  | **β** | ***p*** |  | **β** | ***p*** |
| **Macro architecture** |  |  |  |  |  |  |
| **%REM** |  | -0.176 | 0.518 |  | 0.152 | 0.854 |
| **%N1** |  | 0.044 | 0.794 |  | 0.287 | 0.530 |
| **%N2** |  | 0.041 | 0.932 |  | -1.394 | 0.334 |
| **%N3** |  | 0.090 | 0.851 |  | 0.955 | 0.455 |
| **WASO** |  | -1.747 | 0.340 |  | -4.050 | 0.484 |
| **Micro architecture** |  |  |  |  |  |  |
| **Spindle power** |  | 0.367 | 0.355 |  | -2.111 | 0.027* |
| **Spindle density** |  | -0.005 | 0.868 |  | -0.138 | 0.058 |
| **Spindle frequency** |  | -0.032 | 0.150 |  | -0.116 | 0.065 |
| **Total sleep average ORP** |  | -0.027 | 0.007* |  | -0.069 | 0.008* |
| **N1 average ORP** |  | -0.020 | 0.096 |  | -0.058 | 0.069 |
| **N2 average ORP** |  | -0.026 | 0.015* |  | -0.067 | 0.017* |
| **N3 average ORP** |  | -0.023 | 0.003* |  | -0.046 | 0.012* |
| **REM average ORP** |  | -0.033 | 0.027* |  | -0.072 | 0.106 |
| **Arousal Index** |  | -0.975 | 0.012* |  | -1.718 | 0.086 |

^a^ Covariates include age, sex, race, marital status, educational attainment, parent cohort, probable dementia, hypertension, diabetes, body mass index, total sleep time, apnea-hypopnea index, and the frequency of sleeping pill intake.

^b^ Reference group is *APOE* ε3 homozygotes.

* Denotes statistical significance at the level of *p*<.0.05.

*Acronyms.* APOE=apolipoprotein E; ORP=odds ratio product; REM=rapid eye movement

**Supplementary Table 16.** Sensitivity test of the association between sleep architecture and *APOE* genotype to replacing AHI4% with AHI3%

| **Sleep Characteristics** |  | ***APOE* ε4 Heterozygote** | |  | ***APOE* ε4 Homozygote** | |  | ***APOE* ε2 carriers** | |
| --- | --- | --- | --- | --- | --- | --- | --- | --- | --- |
|  |  | **β** | ***p*** |  | **β** | ***p*** |  | **β** | ***p*** |
| **Macro architecture** |  |  |  |  |  |  |  |  |  |
| **%REM** |  | -0.085 | 0.75 |  | 0.135 | 0.87 |  | 0.022 | 0.94 |
| **%N1** |  | -0.002 | 0.99 |  | 0.304 | 0.51 |  | 0.331 | 0.12 |
| **%N2** |  | -0.063 | 0.89 |  | -1.332 | 0.35 |  | 0.159 | 0.80 |
| **%SWS** |  | 0.149 | 0.75 |  | 0.893 | 0.49 |  | -0.512 | 0.41 |
| **WASO** |  | -1.160 | 0.51 |  | -3.818 | 0.51 |  | -1.086 | 0.59 |
| **Micro architecture** |  |  |  |  |  |  |  |  |  |
| **Spindle power** |  | 0.328 | 0.39 |  | -2.149 | 0.02* |  | -0.089 | 0.85 |
| **Spindle density** |  | -0.008 | 0.77 |  | -0.141 | 0.05 |  | 0.033 | 0.36 |
| **Spindle frequency** |  | -0.044 | 0.04* |  | -0.115 | 0.07 |  | 0.038 | 0.17 |
| **Total sleep average ORP** |  | -0.030 | 0.002* |  | -0.067 | 0.01* |  | 0.005 | 0.71 |
| **N1 average ORP** |  | -0.025 | 0.03* |  | -0.058 | 0.07 |  | 0.002 | 0.91 |
| **N2 average ORP** |  | -0.029 | 0.005* |  | -0.065 | 0.02* |  | -0.0009 | 0.95 |
| **N3 average ORP** |  | -0.022 | 0.002* |  | -0.045 | 0.02* |  | -0.002 | 0.80 |
| **REM average ORP** |  | -0.036 | 0.01* |  | -0.070 | 0.11 |  | 0.004 | 0.83 |
| **Arousal Index** |  | -0.977 | 0.004* |  | -1.324 | 0.17 |  | -0.834 | 0.06 |
| **Total sleep time** |  | -1.467 | 0.59 |  | -3.318 | 0.68 |  | -1.354 | 0.68 |

^a^ Covariates include age, sex, race, marital status, educational attainment, parent cohort, probable dementia, hypertension, diabetes, body mass index, total sleep time, apnea-hypopnea index, and the frequency of sleeping pill intake. For models examining total sleep time and AHI3%, the respective variables were not included as covariates.

^b^ Reference group is *APOE* ε3 homozygotes.

* Denotes statistical significance at the level of *p*<.0.05.

*Acronyms.* AHI3%=apnea-hypopnea index using the 3% desaturation criterion; APOE=apolipoprotein E; ORP=odds ratio product; REM=rapid eye movement
